# Supplementary material for: Assessing the impact of color blindness on the ability of identifying benign and malignant skin lesions by naked-eye examination
Source: PLoS One. 2022 Aug 3;17(8):e0270487. doi: 10.1371/journal.pone.0270487 (PMC9348688; doi:10.1371/journal.pone.0270487)
Supplement: Attachment — Submitted filename: Review.docx [file pone.0270487.s001.docx]

**Review**

**Disclaimer**: I am not a statistician and this manuscript should be reviewed by a statistician

Title: This should include “…by naked-eye examination” because in some countries dermatoscopic evaluation is standard of care for any assessment of pigmented skin lesions by any practitioner

**Design**

What is the duration of a “dermatology rotation” and what proportion of that rotation involves assessing skin lesions with respect to melanoma diagnosis?

Were the readers asked if they were colour-vision impaired and excluded for that reason and were any discovered on Ishihara testing? I ask this because in a cohort of 152 it might be expected that some males at least would have an impairment. I know that does vary with ethnicity but it is worth commenting on.

**Discussion**

I suggest including the following observation in the discussion re Figure 1:

In C the lesion is green and so is the background and in D the lesion is red and the background pink, whereas in B (deuteranope) the surrounding skin is similar to that of the unadjusted image (A)

As a result the colour contrast between the lesion and the surrounding skin is greater for Figure 1B than for C and D which makes irregular structures at the border (right side) more visible (similar as in A). This makes the lesion more clearly asymmetrical which may lean the reader towards a malignant diagnosis.

(The Figure legend should include the appropriate letter for each image respectively)

It would be interesting to see more images. At least a collage similar to Figure 1 but with a malignant lesion. This may or may not support what I have stated about the contrast between lesion and surrounding skin colour. I suggest that the authors evaluate this with every lesion and include those results in the manuscript.

**Additional Limitations**:

The readers were all inexperienced and an undefined proportion were not practising clinicians. There was a selection bias for suspicious lesions which may have influenced the readers (all lesions had been excised). This could in part explain the lower overall accuracy for benign lesions.
