# Supplementary material for: Assessing the impact of color blindness on the ability of identifying benign and malignant skin lesions by naked-eye examination
Source: PLoS One. 2022 Aug 3;17(8):e0270487. doi: 10.1371/journal.pone.0270487 (PMC9348688; doi:10.1371/journal.pone.0270487)
Supplement: Attachment — Submitted filename: Response letter.docx [file pone.0270487.s002.docx]

**Response letter**

Dear editor,

We are glad to submit our revised manuscript entitled:

**Assessing the Impact of Color Blindness on the Ability of Identifying Benign and Malignant Skin Lesions by Naked-Eye Examination**

Journal Requirements:

When submitting your revision, we need you to address these additional requirements.

1. Please ensure that your manuscript meets PLOS ONE's style requirements, including those for file naming. The PLOS ONE style templates can be found at 
<https://journals.plos.org/plosone/s/file?id=wjVg/PLOSOne_formatting_sample_main_body.pdf>and 
<https://journals.plos.org/plosone/s/file?id=ba62/PLOSOne_formatting_sample_title_authors_affiliations.pdf>

**Response**: We reviewed the manuscript and edited it according to journal’s style.

2. We note that Figure 1 in your submission contain copyrighted images. All PLOS content is published under the Creative Commons Attribution License (CC BY 4.0), which means that the manuscript, images, and Supporting Information files will be freely available online, and any third party is permitted to access, download, copy, distribute, and use these materials in any way, even commercially, with proper attribution. For more information, see our copyright guidelines: <http://journals.plos.org/plosone/s/licenses-and-copyright>. We require you to either (1) present written permission from the copyright holder to publish these figures specifically under the CC BY 4.0 license, or (2) remove the figures from your submission:
a) You may seek permission from the original copyright holder of Figure 1 to publish the content specifically under the CC BY 4.0 license. 
We recommend that you contact the original copyright holder with the Content Permission Form (<http://journals.plos.org/plosone/s/file?id=7c09/content-permission-form.pdf>) and the following text:
“I request permission for the open-access journal PLOS ONE to publish XXX under the Creative Commons Attribution License (CCAL) CC BY 4.0 (<http://creativecommons.org/licenses/by/4.0/>). Please be aware that this license allows unrestricted use and distribution, even commercially, by third parties. Please reply and provide explicit written permission to publish XXX under a CC BY license and complete the attached form.”
 
Please upload the completed Content Permission Form or other proof of granted permissions as an "Other" file with your submission. 
 
In the figure caption of the copyrighted figure, please include the following text: “Reprinted from [ref] under a CC BY license, with permission from [name of publisher], original copyright [original copyright year].”
 
b) If you are unable to obtain permission from the original copyright holder to publish these figures under the CC BY 4.0 license or if the copyright holder’s requirements are incompatible with the CC BY 4.0 license, please either i) remove the figure or ii) supply a replacement figure that complies with the CC BY 4.0 license. Please check copyright information on all replacement figures and update the figure caption with source information. If applicable, please specify in the figure caption text when a figure is similar but not identical to the original image and is therefore for illustrative purposes only.

**Response**: We replaced the figure previously used from an open access database with a new one from our patients. We obtained patient’s consent to show the figure and publish it under CC BY 4.0 license.

Additional Editor Comments:
While understanding the impact of color blindness on the ability of diagnosing skin lesions, both clinically and dermoscopically, is certainly of major importance for the dermatology clinic, a study like this is only of use if (1) the right methodology is applied and (2) the data are interpreted without any bias. Thus, in accordance with the expert reviewers I have some conceptual and technical concerns that need to be addressed. Rather than repeat further points here, I refer you to the specific remarks (below) for details.

**Response**: We are thankful for the productive comments provided by the editor and reviewers. We replied to all reviewer’s comments, especially those related to the methodology and data used. We hope our editions and responses were sufficient and satisfy raised concerns.

Reviewers' comments:

Reviewer's Responses to Questions

Comments to the Author

1. Is the manuscript technically sound, and do the data support the conclusions?

The manuscript must describe a technically sound piece of scientific research with data that supports the conclusions. Experiments must have been conducted rigorously, with appropriate controls, replication, and sample sizes. The conclusions must be drawn appropriately based on the data presented.

Reviewer #1: Yes

Reviewer #2: Partly

**Response**: Thank you.

2. Has the statistical analysis been performed appropriately and rigorously?

Reviewer #1: I Don't Know

Reviewer #2: Yes

**Response**: The statistical analysis was performed by an expert biostatistician.

3. Have the authors made all data underlying the findings in their manuscript fully available?

The [PLOS Data policy](http://www.plosone.org/static/policies.action#sharing) requires authors to make all data underlying the findings described in their manuscript fully available without restriction, with rare exception (please refer to the Data Availability Statement in the manuscript PDF file). The data should be provided as part of the manuscript or its supporting information, or deposited to a public repository. For example, in addition to summary statistics, the data points behind means, medians and variance measures should be available. If there are restrictions on publicly sharing data—e.g. participant privacy or use of data from a third party—those must be specified.

Reviewer #1: Yes

Reviewer #2: No

**Response**: The data used in this study is openly and freely accessible at <http://www.cs.rug.nl/~imaging/databases/melanoma_naevi/> with no restriction.

4. Is the manuscript presented in an intelligible fashion and written in standard English?

PLOS ONE does not copyedit accepted manuscripts, so the language in submitted articles must be clear, correct, and unambiguous. Any typographical or grammatical errors should be corrected at revision, so please note any specific errors here.

Reviewer #1: Yes

Reviewer #2: No

**Response**: We performed further English language polishing to ensure the absence of any typographical or grammatical errors.

5. Review Comments to the Author

Please use the space provided to explain your answers to the questions above. You may also include additional comments for the author, including concerns about dual publication, research ethics, or publication ethics. (Please upload your review as an attachment if it exceeds 20,000 characters)

Reviewer #1: Disclaimer: I am not a statistician and this manuscript should be reviewed by a statistician
Title: This should include “…by naked-eye examination” because in some countries dermatoscopic evaluation is standard of care for any assessment of pigmented skin lesions by any practitioner

**Response**: We agree with the reviewer, and we amended the title and further detailed this in the aim accordingly.

Design
What is the duration of a “dermatology rotation” and what proportion of that rotation involves assessing skin lesions with respect to melanoma diagnosis?

**Response**: We provided details on dermatology rotations at Jordanian universities: “all Jordanian universities usually have a 2-week long dermatology rotation with an exam at the end of rotation to assess acquired knowledge.”. Moreover, we provided a link for an optional educational slides on differentiating benign and malignant skin lesions:

<https://reference.medscape.com/features/slideshow/suspicious-skin-lesions>

Were the readers asked if they were colour-vision impaired and excluded for that reason and were any discovered on Ishihara testing? I ask this because in a cohort of 152 it might be expected that some males at least would have an impairment. I know that does vary with ethnicity but it is worth commenting on.

**Response**: We agree with the reviewer, we further explained this in the methodology, where any participant who had an Ishihara score below 12/13 should not proceed with the questionnaire.

Discussion
I suggest including the following observation in the discussion re Figure 1:
In C the lesion is green and so is the background and in D the lesion is red and the background pink, whereas in B (deuteranope) the surrounding skin is similar to that of the unadjusted image (A) As a result the colour contrast between the lesion and the surrounding skin is greater for Figure 1B than for C and D which makes irregular structures at the border (right side) more visible (similar as in A). This makes the lesion more clearly asymmetrical which may lean the reader towards a malignant diagnosis.

**Response**: We would like to than the reviewer for this important observation, which led us to more literature digging to find the basis to explain the observation. We found that the distinction of borders can be affected by colors at the border. However, the distinction is higher for high wavelength colors (i.e., red and green), and the distinction is lower for low-wavelength colors (i.e., blue). We provided this to the discussion to further explain this important point:

“It was noted that the borders between the lesion and the surrounding skin is less distinct for deuteranopia-simulated images, which might increase rater’s suspicion toward malignant lesion. This effect was also observed, but with a lesser degree, for protanopia-simulated images, but not for tritanopia-simulated images. This can be explained by previous studies’ observations, where the distinction of image borders increases for higher wavelength colors (i.e., toward the red colors) [19,20], and deuteranopia- and protanopia-simulated images have more low-wavelength colors than normal or tritanopia-simulated images.”.

(The Figure legend should include the appropriate letter for each image respectively)

**Response**: We added the letters referring to each simulation type.

It would be interesting to see more images. At least a collage similar to Figure 1 but with a malignant lesion. This may or may not support what I have stated about the contrast between lesion and surrounding skin colour. I suggest that the authors evaluate this with every lesion and include those results in the manuscript.

**Response**: We provided detailed discussion on the impact of contrast. As per journal’s request, we had to replace the image used from the database with a one from our own patients in order to publish the image under CC BY 4.0 license, so it was difficult to include an image for a malignant lesion.

Additional Limitations:
The readers were all inexperienced and an undefined proportion were not practising clinicians. There was a selection bias for suspicious lesions which may have influenced the readers (all lesions had been excised). This could in part explain the lower overall accuracy for benign lesions.

**Response**: We agree with the reviewer, and we explained this limitation at the end of discussion as follows:

“Moreover, this study was not performed on specialized dermatologist, whom would have more experience and confidence in diagnosing benign lesions as benign depending on more lesion characteristic, other than color. Further studies in this regard might show if including experienced dermatologist might abolish such accuracy difference for participants with color vision deficiency.”

Reviewer #2: Dear Authors,

thank you for your manuscript "Assessing the impact of color blindness on the ability of identifying benign and malignant skin lesions". The idea behind your study is appreciated. However, there are some important issues to be adressed.
1) please improve the writing, there are many errors to be corrected (example: Page 1. Background: Color vision deficiency describes the inability to distinguish certain
shades of color. We found a significantlyhigher mean accuracyasthe accuracy of classifying)

**Response**: We reviewed the manuscript for typographical or grammatical errors. The above-mentioned typographical error appeared due to pdf-HTML conversion by the editorial system.

2) diagnostic precision. You cite several sources to emphasize that "The diagnosis and referral accuracy of pigmented skin lesions are generally low among primary care physicians compared to dermatologists [12–14]." Despite that fact your study was conducted with participant in their final year in medical school or newly graduated. Please describe your intention to choose this group of participants and discuss a potential impact on the results.

**Response**: This is an important point to elaborate. We explained the rationale of including participant in their final year in medical school or newly graduated in the introduction as follows: “We conducted this study on participant in their final year in medical school or newly graduated, whom we believe depends more on lesion color criteria in distinguishing benign and malignant lesions. Moreover, it also enabled us to include a relatively larger sample size.”. However, including such sample resulted in a limitation compared to including experienced dermatologist, which we also detailed in study limitations as follows: “Moreover, this study was not performed on specialized dermatologist, whom would have more experience and confidence in diagnosing benign lesions as benign depending on more lesion characteristic, other than color. Further studies in this regard might show if including experienced dermatologist might abolish such accuracy difference for participants with color vision deficiency.”.

3) "we used Vischeck color blindness simulator in Fiji software [16], which has been proved to be highly accurate in simulating color-blind images [17]".
It remains unclear how the whole process was conducted. Please specify how the images were presented (probably online via a private account shown on an uncertified screen). Especially the presentation of colors may vary and is dependent from many factors (model and age of the screen, contrast, intensity, surroundings etc). This might be crucial regarding the results.

**Response**: We agree with the reviewer on the importance of elaboration on this part. We used Google forms for data collection. To account for device variability, we assessed data about skin lesion classification without simulation, in order to compare with classification accuracy for simulated images. However, as this does not eliminate the impact of device screen variability, we also added a limitation statement in this regard: “Another limitation to keep in mind is that participants used their own devices to complete the questionnaire, which might result in impact of screen color characteristic and brightness on classification accuracy.”

Best regards and good luck!

6. PLOS authors have the option to publish the peer review history of their article ([what does this mean?](https://journals.plos.org/plosone/s/editorial-and-peer-review-process#loc-peer-review-history)). If published, this will include your full peer review and any attached files.

If you choose “no”, your identity will remain anonymous but your review may still be made public.


Do you want your identity to be public for this peer review? For information about this choice, including consent withdrawal, please see our [Privacy Policy](https://www.plos.org/privacy-policy).

Reviewer #1: No

Reviewer #2: No

While revising your submission, please upload your figure files to the Preflight Analysis and Conversion Engine (PACE) digital diagnostic tool, <https://pacev2.apexcovantage.com/>. PACE helps ensure that figures meet PLOS requirements. To use PACE, you must first register as a user. Registration is free. Then, login and navigate to the UPLOAD tab, where you will find detailed instructions on how to use the tool. If you encounter any issues or have any questions when using PACE, please email PLOS at [figures@plos.org](mailto:figures@plos.org). Please note that Supporting Information files do not need this step.
