# Supplementary material for: Assessing the impact of color blindness on the ability of identifying benign and malignant skin lesions by naked-eye examination
Source: PLoS One. 2022 Aug 3;17(8):e0270487. doi: 10.1371/journal.pone.0270487 (PMC9348688; doi:10.1371/journal.pone.0270487)
Supplement: Attachment — Submitted filename: Review R1.docx [file pone.0270487.s003.docx]

**Review R1**

Page 16: The following confusing statement must be clarified:

“We calculated the score out of 5 for each of benign and malignant lesion images

correctly identified in each of the four sets and calculated the accuracy score as a percentage for each.” At face value this states that each image is scored out of 5 so there would be a maximum score of 50 for each set of 10 images.

The next sentence, on face value, describes a second calculation.

This does not make sense to me.

Page 18: The following sentence does not make sense:

Table 2 details the mean accuracy of diagnosing benign and malignant lesions for each group the difference in accuracy between benign and malignant lesion classification. (the same sentence is repeated in the legend for Table 2.

Table 2 presents the same data as Table 1 for benign and malignant accuracy, including values and Standard Deviation and essentially just adds a decimal point to the values and adds p-value, Mean Difference and 95% CI. This could be presented in a single table. An inconsistency between the two tables for malignant lesion accuracy for Deuteranope images is noted (80.6% vs. 82.58%)

The legend of Table 3 is not a complete sentence. I actually see no purpose in including Table 3 at all. The preceding sentence conveys the negative findings adequately.

**Discussion**

Page 20: There is a major typo: This study tested the accuracy of diagnosing benign and malignant pigmented skin lesions among simulated images as if seen by ***patients*** with different types…

Re this statement: “Upon performing our sub-group analysis to analyze if there was a difference in diagnosis accuracy for benign or malignant lesions, we found that the diagnostic accuracy for benign lesions were significantly lower for all images, with highest magnitude for deuteranopia simulated images” it is actually noted that non-simulated images were associated with a lower accuracy for benign lesions than both simulated groups except Deuteranope. This must be acknowledged and discussed.

**Conclusion**

The study was not on distinguishing skin lesions, as stated , but on distinguishing images of skin lesions. That is significant and must be stated in the conclusion.

The images presented in the revised manuscript (Figure 1) bear no reasonable resemblance at all to any skin lesion, benign or malignant.

**English Grammar Issues**

Page 12: Classification accuracy for malignant lesions ***were*** higher than…

Page 14: We conducted this study on participants in their final year in medical

school or newly graduated, whom we believe ***depends*** more on lesion color …

The study included participants who were either ***at*** their final year in medical school or newly graduated and ~~were~~ working as general practitioners.

Page 15: Using an online form, the nature of the ***project described*** and a consent to participate signed

Page 16: In order to correct for device’s screen variabilities, we compared the ***resulted***

accuracy for ***none simulated*** images with simulated images.

(Fig 1) shows a benign skin lesion simulated into what a protanope, deuteranope, and tritanope ***participants*** can see. The 40 images were shuffled and ***presented sequentially each image alone.***

Page 18: Upon comparing classification accuracy for benign and malignant lesions between each image simulation, we found significant differences for all images, where classification accuracy for malignant lesions ***were*** higher than classification accuracy for benign lesions

Page 20: Upon performing our sub-group analysis to analyze if there was a difference in

diagnosis accuracy for benign or malignant lesions, we found that the diagnostic accuracy for

benign lesions ***were*** significantly lower for all images, with highest magnitude for deuteranopia simulated images

Page 21: Many efforts ***has*** been evaluated…

Page 22: …it still has limitations that need to be considered ***upon result’s*** interpretation

Page 22: clinical decision to decide ***to wither*** a lesion is benign or…

Page 22: Moreover, this study was not ***performed on specialized dermatologist***, ***whom*** would have more experience and confidence in diagnosing benign lesions as benign depending on ***more lesion characteristic***, other than color

Page 22: Further studies in this regard might show if including experienced ***dermatologist*** might
