# Supplementary material for: Assessing the impact of color blindness on the ability of identifying benign and malignant skin lesions by naked-eye examination
Source: PLoS One. 2022 Aug 3;17(8):e0270487. doi: 10.1371/journal.pone.0270487 (PMC9348688; doi:10.1371/journal.pone.0270487)
Supplement: Attachment — Submitted filename: Response to Reviewers.docx [file pone.0270487.s004.docx]

**Response letter**

Dear editor,

We are glad to submit our revised manuscript entitled:

**Assessing the Impact of Color Blindness on the Ability of Identifying Benign and Malignant Skin Lesions by Naked-Eye Examination**

**Reviewer #1:**

Page 16: The following confusing statement must be clarified:

“We calculated the score out of 5 for each of benign and malignant lesion images

correctly identified in each of the four sets and calculated the accuracy score as a percentage for each.” At face value this states that each image is scored out of 5 so there would be a maximum score of 50 for each set of 10 images.

The next sentence, on face value, describes a second calculation.

This does not make sense to me.

**Reply**: We agree with the reviewer that the statement was not clear, so we rewrote the statement as follows:

“Each image’s answer was scored as either correct (1) or incorrect (0). There were four sets of images, each containing images for five benign and five malignant lesions. Of the four sets, 3 were simulated into into what a deuteranope, protanope, and tritanope participan can see, and the last set was not simulated. We calculated accuracy score as a percentage for the benign and malignant images in each set, then accuracy of image classification in the set (aka., overall accuracy).”

Page 18: The following sentence does not make sense:

Table 2 details the mean accuracy of diagnosing benign and malignant lesions for each group the difference in accuracy between benign and malignant lesion classification. (the same sentence is repeated in the legend for Table 2.

**Response**: We rewrote the statement and figure legend to be better descriptive for the results:

“the mean accuracy of classifying lesions presented in the images in each set (i.e., protanope, deuteranope, tritanope simulated images and non-simulated images) as either benign or malignant lesions. It also present the mean difference in classification accuracy between images containing benign and malignant lesions.”

Table 2 presents the same data as Table 1 for benign and malignant accuracy, including values and Standard Deviation and essentially just adds a decimal point to the values and adds p-value, Mean Difference and 95% CI. This could be presented in a single table. An inconsistency between the two tables for malignant lesion accuracy for Deuteranope images is noted (80.6% vs. 82.58%)

**Response**: Thank you for the suggestion. We combined both tables into one as suggested and rechecked the numbers presented for accuracy.

The legend of Table 3 is not a complete sentence. I actually see no purpose in including Table 3 at all. The preceding sentence conveys the negative findings adequately.

**Response**: We completed the figure legend. Despite the absence of significant results in the table, we think it provides important descriptive data to be considered in gender comparison.

**Discussion**

Page 20: There is a major typo: This study tested the accuracy of diagnosing benign and malignant pigmented skin lesions among simulated images as if seen by ***patients*** with different types…

**Response**: Corrected, thank you.

Re this statement: “Upon performing our sub-group analysis to analyze if there was a difference in diagnosis accuracy for benign or malignant lesions, we found that the diagnostic accuracy for benign lesions were significantly lower for all images, with highest magnitude for deuteranopia simulated images” it is actually noted that non-simulated images were associated with a lower accuracy for benign lesions than both simulated groups except Deuteranope. This must be acknowledged and discussed.

**Response**: Thank you for this observation. We acknowledged this finding, however, it might be difficult to further elaborate on it due to the small difference in benign lesion diagnosis accuracy between non-simulated, and those protanopia and tritanopia simulated images.

**Conclusion**

The study was not on distinguishing skin lesions, as stated , but on distinguishing images of skin lesions. That is significant and must be stated in the conclusion.

**Response**: We edited the conclusion and the manuscript throughout to ensure correct wording used.

The images presented in the revised manuscript (Figure 1) bear no reasonable resemblance at all to any skin lesion, benign or malignant.

**Response**: The database containing images used in the survey was provided in its respective database: <http://www.cs.rug.nl/~imaging/databases/melanoma_naevi/>

Due to copyright for the database, we can’t use any of its images as a figure. We obtained images for a benign (as in the figure 1), and for a malignant lesion (new figure 2) to show how simulation works. We agree with the reviewer that using images from the original database might be more reflective to what we used in this study, however, we could not obtain copyrights for database’s images.

**English Grammar Issues**

Page 12: Classification accuracy for malignant lesions ***were*** higher than…

Page 14: We conducted this study on participants in their final year in medical

school or newly graduated, whom we believe ***depends*** more on lesion color …

The study included participants who were either ***at*** their final year in medical school or newly graduated and ~~were~~ working as general practitioners.

Page 15: Using an online form, the nature of the ***project described*** and a consent to participate signed

Page 16: In order to correct for device’s screen variabilities, we compared the ***resulted***

accuracy for ***none simulated*** images with simulated images.

(Fig 1) shows a benign skin lesion simulated into what a protanope, deuteranope, and tritanope ***participants*** can see. The 40 images were shuffled and ***presented sequentially each image alone.***

Page 18: Upon comparing classification accuracy for benign and malignant lesions between each image simulation, we found significant differences for all images, where classification accuracy for malignant lesions ***were*** higher than classification accuracy for benign lesions

Page 20: Upon performing our sub-group analysis to analyze if there was a difference in

diagnosis accuracy for benign or malignant lesions, we found that the diagnostic accuracy for

benign lesions ***were*** significantly lower for all images, with highest magnitude for deuteranopia simulated images

Page 21: Many efforts ***has*** been evaluated…

Page 22: …it still has limitations that need to be considered ***upon result’s*** interpretation

Page 22: clinical decision to decide ***to wither*** a lesion is benign or…

Page 22: Moreover, this study was not ***performed on specialized dermatologist***, ***whom*** would have more experience and confidence in diagnosing benign lesions as benign depending on ***more lesion characteristic***, other than color

Page 22: Further studies in this regard might show if including experienced ***dermatologist*** might

**Response**: Thank you for going through the manuscript for English language. We went through the manuscript to correct the pointed mistakes and other missed ones.

**Reviewer #2:**

Thank you for improving your manuscript significantly. "We conducted this study on participant in their final year in medical school or newly graduated, whom we believe depends more on lesion color criteria in distinguishing benign and malignant lesions. Moreover, it also enabled us to include a relatively larger sample size." As far as I know the first statement is a hypothesis. The motivation to include a larger sample size might not justify to conduct such a study with graduating medical students instead of general practicioners or dermatolists. From a clinical stanpoint, conducting this study with general pracitioners would probably be ideal in order to simulate what you wanted to express.

**Response**: Thank you for this important point. We edited the manuscript accordingly.
