# Supplementary material for: Assessing the impact of color blindness on the ability of identifying benign and malignant skin lesions by naked-eye examination
Source: PLoS One. 2022 Aug 3;17(8):e0270487. doi: 10.1371/journal.pone.0270487 (PMC9348688; doi:10.1371/journal.pone.0270487)
Supplement: Attachment — Submitted filename: Response to Reviewers.docx [file pone.0270487.s005.docx]

**Response letter**

Dear editor,

We are glad to submit our revised manuscript entitled:

**Assessing the Impact of Color Blindness on the Ability of Identifying Benign and Malignant Skin Lesions by Naked-Eye Examination**

Thank you for submitting your manuscript to PLOS ONE. After careful consideration, we feel that it has merit but does not fully meet PLOS ONE’s publication criteria as it currently stands. Therefore, we invite you to submit a revised version of the manuscript that addresses the points raised during the review process.

I'd like to thank the authors for improving the manuscript. However, there are still some points that need to be addressed:

(1) The point of the study is to assess the impact of colour deficiency on diagnosis of photos of pigmented skin lesions. Therefore, Figures 1 and 2 should be representative of the examined dataset and should thus be replaced with better examples (clinically). Secondly, the quality should be similar or better than that of the Figure in the original submission (the image quality of the new images is not sufficient), unless the quality of the actual dataset was similarly low as that of the new figures. The figure legend should contain the given diagnosis (e.g. compound naevus and superficial spreading melanoma). Moreover, and importantly, it should be made clear in text and legend that the lesions in Figures 1 and 2 are NOT included in the set of assessed lesions.

**Reply**: Thank you for the clearly explained comment. We the main issue in the original image was the copyright of its deposition did not meet the copyright requirements of the Plos One journal, so we could not include one of the images used in the dataset as a figure in the manuscript. We made this point clear as requested in the figure legend. We discarded the previously included Fig.1 due to its low quality and we replaced it with a better figure with an improved quality and representation of the original dataset. The malignant lesion has similar characteristics to the images present within the MED_NOD dataset, so we just worked to improve its quality to meet journal’s requirements.

(2) ‘Each image’s answer’ should be corrected to ‘The diagnosis for each image’

**Response**: Done, thank you.

(3) ‘aka. overall accuracy’ should be corrected to ‘i.e. overall accuracy’

**Response**: Done, thank you.

(4) Table 2 should state ‘between males and female participants’ rather than ‘between males and females’

**Response**: Done, thank you.

(5) ‘rater’s suspicion toward malignant’ should be ‘rater’s bias toward malignant’

**Response**: Done, thank you.

(6) There are still multiple semantic and/or grammatical errors.

**Response**: We sent the manuscript for a professional medical English language professional.
