# Supplementary material for: Assessing the impact of color blindness on the ability of identifying benign and malignant skin lesions by naked-eye examination
Source: PLoS One. 2022 Aug 3;17(8):e0270487. doi: 10.1371/journal.pone.0270487 (PMC9348688; doi:10.1371/journal.pone.0270487)
Supplement: Attachment — Submitted filename: Response to Reviewers.docx [file pone.0270487.s006.docx]

**Response letter**

Dear editor,

We are glad to submit our revised manuscript entitled:

**Assessing the Impact of Color Blindness on the Ability of Identifying Benign and Malignant Skin Lesions by Naked-Eye Examination**

Thank you very much for sending us the original sets of images used in the study. These photos certainly made a difference to the reviewers and me. In fact, I had two lengthy phone conversations with the reviewers today. As you can imagine from our previous communication, it would still be best to publish all 40 images of the test set as supplementary data, as this would allow not only us but also the reader to assess them and therefore make the paper more valuable to the community. Thus, I would encourage you (possibly together with the PLOS ONE editorial team) to have another strong attempt to get permission from the source of the photographs to publish them as supplementary information. If this is not possible, you should make a clear statement in the paper that the photographs are available from you on request at any time (the same way as you provided them to us privately). In any case, you will need to change the figures in the manuscript, as the point of the study is to assess the impact of colour deficiency on diagnosis of photos of pigmented skin lesions. Therefore, Figures 1 and 2 should be similar to the examined dataset and should thus be replaced with better examples. Figure 1 (benign lesion) looks reasonably similar to the benign lesions in the test sets. However, Figure 2 (melanoma) is structurally so different from Figure 1 that even a black-and-white image would suffice to see the difference (i.e. the difference between Figure 1 and 2 in the current version of the manuscript PONE-D-21-20492R3 is not relevant for the message of the study). Hence, a melanoma that is more similar to a naevus or a naevus that is more similar to a melanoma should be picked for illustration in Figures 1 and 2, to reflect the more subtle colour differences (relevant for colour deficient assessors) between benign and malignant lesions, rather than the less colour-dependent structural differences. Moreover, and importantly, it should be made clear in text and legend that the lesions in Figures 1 and 2 are NOT included in the set of assessed lesions.

**Reply**:

We highly appreciate the time and effort Prof. Haass invested in our manuscript.

First, we deposited the images used in this study, including simulated images, in a separate database and we provided citation and link to the database:

AlRyalat, Saif Aldeen, 2022, "Cutaneous benign and malignant lesions simulated for color blindness", <https://doi.org/10.7910/DVN/OX324U>, Harvard Dataverse, V1

Second, we replaced figure two, where we used a new image for a melanoma previously reported in a Plos One article:

“Longo C, Piana S, Lallas A, Moscarella E, Lombardi M, Raucci M, Pellacani G, Argenziano G. Routine clinical-pathologic correlation of pigmented skin tumors can influence patient management. PLoS One. 2015 Sep 1;10(9):e0136031”

This image has a CC-BY copyright license. We used this image and we provided proper citation accordingly.

Third, we reiterated on the fact that the figures presented in this article (figures 1 and 2) were not used as part of the survey used for data collection.

We hope these changes satisfy the merit for publication in Plos One Journal.
